# Stem Cell-Based or Cell-Free Gene Therapy in Chondrocyte Regeneration: Synovial Fluid-Derived Mesenchymal Stem Cell Exosomes

**DOI:** 10.2174/0115665240266016231014081916

**Published:** 2023-10-19

**Authors:** Onur Uysal, Haya Erybeh, Mediha Canbek, Emilia Qomi Ekenel, Sibel Gunes, Gülay Büyükköroğlu, Tugba Semerci Sevimli, Fatih Cemrek, Ayla Eker Sariboyaci

**Affiliations:** 1 Cellular Therapy and Stem Cell Production Application and Research Centre, ESTEM, Eskisehir Osmangazi University, Eskisehir, Turkiye;; 2 Department of Stem Cell, Institute of Health Sciences, Eskisehir Osmangazi University, Eskisehir, Turkiye;; 3 Department of Medical Laboratory Techniques, Vocational School of Health Services, Eskisehir, Turkiye;; 4 Department of Molecular Biology, Science Faculty, Eskisehir Osmangazi University, Eskisehir, Turkiye;; 5 Department of Pharmaceutical Biotechnology, Faculty of Pharmacy, Anadolu University, Eskisehir, Turkiye;; 6 Department of Statistics, Faculty of Science and Letters, Eskisehir Osmangazi University, Eskisehir, Turkiye

**Keywords:** Cartilage defects, synovial fluid mesenchymal stem cells, exosomes, chondrogenesis, hsa-miR-155-5p, fetal human chondroblast cell line

## Abstract

**Background:**

Cartilage injuries are currently the most prevalent joint disease. Previous studies have emphasized the use of stem cells as the effective treatment for regenerating cartilage damage.

**Objective:**

In this study, considering the difficulties of the cellular therapy method, it was hypothesized that human synovial fluid-derived mesenchymal stem cell (hSF-MSC) exosomes as a SC source could be used to treat these injuries as a safer and cell-free therapeutic alternative procedure due to its direct relevance to cartilage regeneration. Moreover, this study aimed to determine the miRNA and target genes required for the formation of SC treatment combined with gene therapy in order to reveal the mechanism of cartilage regeneration and increase its effectiveness.

**Methods:**

MSCs were characterized by flow cytometry, and immunocytochemical and differentiation analyses were done. To characterize functionally isolated exosomes, *in vitro* uptake analysis was performed. RT-qPCR was used to examine in terms of the advantages of cellular and cell-free therapy, mature human chondroblasts derived by differentiation from hSF-MSCs and human chondrocyte profiles were compared in order to demonstrate the above profile of hSF-MSCs and exosomes isolated from them, and the effectiveness of SC therapy in repairing cartilage damage.

**Results:**

According to our findings, the expression level of hsa-miR-155-5p was found to be considerably higher in chondrocytes differentiated from human synovial fluid MSCs than in mature human chondrocytes. These findings were also supported by the TGF-signalling pathway and chondrogenesis marker genes.

**Conclusion:**

It was concluded that hSF-MSCs and exosomes can be used in the treatment of cartilage damage, and hsa-miR-155-5p can be used as a target miRNA in a new gene therapy approach because it increases the therapeutic effect on cartilage damage.

## 1 INTRODUCTION

The most prevalent degenerative joint disorders that impact the majority of the world's population, cause morbidity, and impair joint function are osteoarthritis and full-thickness cartilage abnormalities [1]. The use of adult stem cells is one of the most often used clinical strategies in the treatment of cartilage injury [2].

While the results from the cellular therapy approach in the treatment of cartilage damage are promising, several challenges have been observed, of these naturally occurring approaches, which may pose some risks in clinical practice including the heterogeneity of proliferating cells *in vitro* [3]. Numerous researches have shown that the paracrine theory, which is what motivates the development of a new cell-free alternative method in regenerative medicine, is correct that the therapeutic impact demonstrated by MSCs is connected to their secretions [4, 5]. The utilization of exosomes produced by mesenchymal stem cells attracts attention as a less dangerous alternative to mesenchymal stem cell transplantation [6-8]. Exosomes that are lipid bilayer vesicles released by multiple cell types have been emerged as a popular “cell-free” alternative therapeutics and can be replace cellular therapeutic agents to overcome their risky challenges [7, 9, 10]. A safer cell-free therapeutic option to stem cell transplantation, the use of exosomes derived from MSCs to treat cartilage injuries has garnered attention; nonetheless, studies on exosomes are restricted and still require further research. Exosomes are produced when multivesicular bodies created by the endosomal pathway fuse with the plasma membrane. By moving through the bodily fluids or making contact with the nearby target cell, exosomes can be released to target distant cells [11]. Exosomes' role in facilitating intercellular communication by exchanging proteins and genetic information is one of its key activities. It is also well known that the mRNA and miRNA composition of exosomes can control the receiving cells' ability to synthesize proteins. There are few studies that show miRNAs are involved in chondrogenesis, and their molecular processes are not fully understood [12-15]. Many studies have demonstrated the role of some miRNAs in human MSCs functions. For example, it has been shown that hsa-miR-155-5p regulates the immunoregulatory properties of MSCs [16, 17]. Moreover, TGF-β, plays a role in chondrocyte differentiation *via* its intracellular SMADs signaling pathway, [15, 18, 19]. Given this background, according to our literature research, to the best of our knowledge no study has yet investigated the expression profile of hsa-miR-155-5p in synovial fluid-derived MSCs along with their exosomes, nor its role in the chondrogenesis process. Therefore, the purpose of this study was to explore the function of hsa-miR-155-5p in the chondrogenesis process. The results of our study may reveal a new cell-free clinical approach in the treatment of articular cartilage damage and may illuminate part of the molecular mechanism for the role of hsa-miR-155-5p in chondrogenesis. Because of this, in the first part of this study comprise the expression profiles of hsa-miR-155-5p in the chondrogenesis and regeneration of damaged cartilage tissue, genes in TGF-β signaling pathway into chondrogenic differentiation of SMAD2, SMAD3, and SMAD4 genes as well as chondrogenesis markers, including Aggrecan (ACAN) and Collagen IA2 (COL1A2) in human synovial fluid-derived from MSCs and exosomes isolated from them were analysed by RT-qPCR. In the second part of the study, in order to demonstrate the effectiveness of stem cell therapy in the repair of cartilage damage, chondroblasts obtained by differentiation from human synovial fluid derived from MSCs and also mature human chondrocytes together with the same miRNA and genes were analysed.

## 2 MATERIALS AND METHODS

In this study, human knee synovial fluid-derived mesenchymal stem cells (hSF-MSCs) were used. They were isolated after informed consent from three healthy volunteers, with approval of the ethical board and Informed consent was obtained from the participants. The study proceeded with the approval of the ethical board of the Eskisehir Osmangazi University, Medical Faculty, Health Practice and Research Hospital, Clinical Research Ethics Committee (Protocol No: 11.04.2019/20), characterized and cryopreserved to be used in future experiments. Briefly, synovial fluid samples were passed through a 100 µm and 70 µm cell strainer (pluri Strainer, Leipzig, Germany), respectively. It was then centrifuged at 300 g for 5 minutes and resuspended with complete medium containing 15 mL of 10% fetal bovine serum (FBS; Invitrogen, Gibco, Göteborg, Sweden), 0.2% primocin (Invivogen, Toulouse, France), 1% (ml/ml) Glutamax (Sigma Aldrich, Buchs, Switzerland). It was cultured in an incubator (Panasonic MCO-170M-PE, Kadoma, Japan) at 37°C with 5% O_2_, 5% CO_2_ and 95% humidity. After the cells adhered, the medium was changed every 2-3 days, and when they reached 70-80% confluency, they were split at a ratio of 1:3 and passaged.

### 2.1 Thawing and Culture of hSF-MSCs

**Fig. (1):**
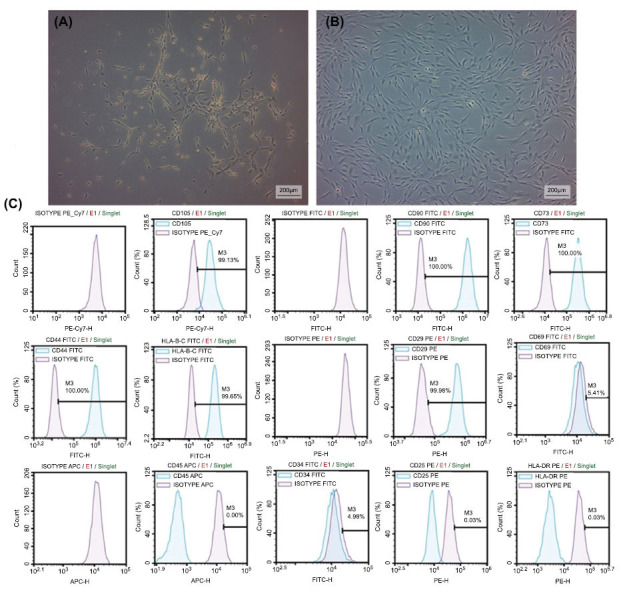
Morphology of hSF-MSCs at phase-contrast microscopic images and representative flow cytometry analysis of cell surface markers on hSF-MSCs. (**A**): Fibroblast like morphology of hSF-MSCs. (**B**): Confluent growth of mesenchymal stem cells in P3 (Scale bars: 200 µm). (**C**): Representative flow cytometry analysis of cell-surface markers; CD105, CD90, CD73, CD44, MHC I, CD29, CD69, CD45, CD34, CD25 and MHC II on hSF-MSCs at P3.

Cells were thawed and cultured in a complete DMEM F12 culture medium (Gibco) containing 10% fetal bovine serum (FBS; Gibco) and 0.2% primocin (Invivogen) (Fig. **1A** and **B**). Isolated cells were incubated at 37°C in a humidified atmosphere containing 5% CO_2_ and 5% O_2_ as previously described [20]. Microscopic examinations were performed on a daily basis, and the culture medium was replaced every three days following cell adherence. Once the cells attained 70-80% confluence, they were sub-cultured by detaching them from the culture dish using trypsin-EDTA (Gibco) and then seeding them into a new culture dish at a 1:3 dilution ratio. Cells cultured to passage 3 (P3) by subculturing were used in the characterization analysis.

### 2.2 Characterization of hSF-MSCs

Cell phenotypic characteristics analyses were done to confirm that hSF-MSCs maintain them after cryopreservation, MSCs were analysed by flow cytometry, and immunocytochemical and differentiation (adipogenic, osteogenic, and chondrogenic) analyses were done.

#### 2.2.1 Immunophenotypical Characterization of hSF-MCSs

##### 2.2.1.1 Flow Cytometry Analysis of hSF-MSCs

To assess the stemness of isolated hSF-MSC, antibodies against mesenchymal stem cell surface markers were used (Table **1**). CD105, CD90, CD73, CD44, MHC I, CD29, CD69, CD45, CD34, CD25, and MHC II conjugated-antibodies (Biolegend, London, UK) were used for the hSF-MSC’s characterization (Table **1**).

Antibody staining was performed according to the manufacturer's protocol. Briefly, cells (1x10^6^ viable cells/mL) were incubated with 1:100 diluted antibodies at 4°C in the dark. After the incubation, stained cells are injected into the flow cytometer device. Flow cytometry was performed using a Novocyte D3005 standard configuration (Agilent, Santa Clara, USA). The data were analysed with NovoExpress flow cytometry software (version 1.5.0, Agilent). All flow cytometry analysis was performed on hSF-MSCs at P3.

##### 2.2.1.2 Immunocytochemical Analysis of hSF-MSCs

**Fig. (2):**
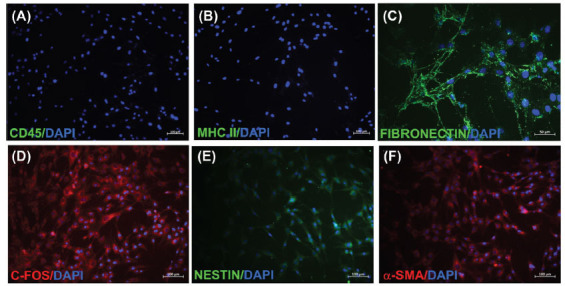
Representative panels of immunofluorescence in hSF-MSCs. hSF-MSCs were negative for CD45 (green) (**A**), MHC class II (green) (**B**) and positive for the FIBRONECTIN (green) (**C**), C-FOS (red) (**D**), NESTIN (green) (**E**) and α-SMA (red) (**F**). Nuclei were stained with DAPI (blue) (**A**-**F**) (Scale bars: 100, 100, 50, 100, 100 and 100 μm).

To identify the cell surface markers of isolated hSF-MSCs, cells from the third passage were then taken for immunofluorescence labelling (IF), as described in Table **1** and (Fig. **2**) [21]. IF staining of cells was performed as in our previous study [22]. Cells were fixed in ice-cold methanol. After the permeabilization stage with 0.025% Triton X-100 (Merck, Taufkirchen, Germany), the cells were incubated with 1.5% normal goat or donkey blocking serum (Santa Cruz Biotechnology, Heidelberg, Germany) in PBS (Gibco™) to suppress non-specific binding of IgG. After washing, the cells were incubated overnight with primary antibodies (CD45, MHC class II, FIBRONECTIN, C-FOS, NESTIN, and α-SMA) (Biolegend). After that, the cells were incubated with appropriate fluorescein isothiocyanate (FITC)- and Texas Red-labelled secondary antibodies (Santa Cruz). After washing, the cells were mounted with medium containing 4',6- diamidino-2-phenylindole (DAPI, Santa Cruz). Microscopic analysis and images were taken using a fluorescence microscope (DM 2500; Leica, Wetzlar, Germany).

##### 2.2.1.3 *In vitro* Differentiation Analysis of hSF-MSCs for Characterization

*In vitro* differentiation studies of adipogenic, osteogenic, and chondrogenic differentiations were performed according to the methods described previously [21, 23, 24]. Three technical replicates of each sample were analyzed.

##### 2.2.1.4 Adipogenic Differentiation

Passage three cells, seeded at a density of 3000 cells/cm^2^, were cultured on type I collagen-coated coverslips (BD Biosciences, CA, USA) in 6-well plates to induce adipogenic differentiation. Adipogenic differentiation was induced by incubating the cells in DMEM (Gibco) containing 10% FBS (Invitrogen, Gibco), 0.5 mM isobutyl-methylxanthine (IBMX, Sigma Aldrich), 10^6^ M dexamethasone (Sigma Aldrich), 10 mg/ml insulin (Invitrogen), 200 mM indomethacin (Sigma-Aldrich), and 0.2% primocin (Invivogen) for four weeks. The culture medium was replaced twice per week. Adipogenic differentiation was confirmed by the presence of intracellular lipid droplets, which were detected by histochemical staining with Oil Red O (ORO) (Sigma Aldrich) and immunocytochemical labeling with ADIPONECTIN (ADN) (Sigma Aldrich). Cytoskeleton was labelled with ACTIN (Sigma Aldrich) to nonspecifically visualize the cell cytoplasm and demonstrate that lipid vacuoles are localized intra- or intercellular stained with ORO.

##### 2.2.1.5 Osteogenic Differentiation

Cells from passage three (3,000 cells/cm^2^) were seeded onto 6-well plates with type I collagen coated coverslips. For osteogenic differentiation, the cells were incubated in DMEM (Gibco) containing with 100 nM dexamethasone (Sigma-Aldrich), 0.05 mM ascorbate-2-phosphate (Wako Chemicals, Richmond, USA), 10 mM glycerophosphate (Sigma-Aldrich), 0.2% primocin (Invivogen), and 10% FBS (Invitrogen, Gibco). The differentiation medium was replaced twice a week. At the end of the fourth week, osteogenic differentiation was evaluated by histochemical staining with Alizarin Red S, which stains calcium deposits (ARS; Sigma Aldrich Switzerland) and immunocytochemical labelling with OSTEOCALCIN (OCN) (Sigma Aldrich).

##### 2.2.1.6 Chondrogenic Differentiation

In order to induce chondrogenic differentiation, 2.5x10^5^ cells were seeded into a 15 mL polypropylene tube and centrifuged at 1300xg for 5 minutes to form a pelleted micromass in 3D culture. The micromass was then cultured in chondrogenic medium for two weeks, with medium changes occurring twice per week. The chondrogenic medium consisted of high glucose DMEM (Gibco) supplemented with 10 ng/mL transforming growth factor-β1 (TGF-β1; Sigma Aldrich), 50 µg/mL ascorbate-2-phosphate (Wako Chemicals), 0.1 µM dexamethasone (Sigma Aldrich), 100 µg/ml sodium pyruvate (Sigma Aldrich), 40 µg/mL proline (Merck), 50 mg/ml ITS premix (BD Biosciences), and 0.2% primocin (Invivogen). The resulting pellets were fixed with 4% paraformaldehyde, embedded in paraffin, and then subjected to histochemical staining with Alcian Blue (AB, Sigma Aldrich) and Safranin O (SO, Sigma Aldrich), which stain glycosaminoglycans, as well as immunohistochemical labeling with COLLAGEN II (COLL II, Sigma Aldrich) to assess chondrogenic differentiation.

### 2.3 Exosome Isolation, Purification and Characterization

In this study, exosomes were first isolated from MSCs using an exosome isolation kit (Invitrogen). Exosomes were then purified by tangential ultrafiltration and diafiltration with the tangential flow filtration (TFF) system (Sartorius, Sartoflow Smart, Goettingen, Germany) to eliminate co-isolating microvesicles. For human synovial fluid-derived mesenchymal stem cell exosome (hSF-MSC-Exo) isolation, cells were seeded in a 75 cm^2^ flask and left to reach 70% confluency. Exosomes were isolated using a Total Exosome Isolation Reagent Kit (from cell culture media) (Invitrogen, Göteborg, Sweden) and according to the manufacturer's protocol. To purify the isolated exosomes, they were purified by cross-flow ultrafiltration and diafiltration with a tangential flow filtration (TFF) system (Sartorius, Sartoflow Smart, Goettingen, Germany) containing a fiber cartridge (Sartorius, Goettingen, Germany) with a cut-off value of 100 kDa. Nanomembrane concentrators Vivaspin 20, 100 kDa MWCO (Sartorious) were washed to remove glycerol and other preservatives by adding 20 ml of PBS buffer and then centrifuged for 30 min at 3,000xg at 25°C before processing exosome samples. Exosomes larger than 100 kDa were eliminated by filtration and the sample was concentrated approximately 100x. Purified exosomes will be stored at -80°C for long-term storage.

Flow cytometry analysis was carried out to characterize isolated exosomes using certain surface markers. 3 μm diameter beads were used for flow cytometry analysis. CD63 (Invitrogen) antibody was used to coat the beads first. Then the coated beads were incubated with isolated exosomes. They were treated with CD81 (PE, Invitrogen) and CD9 (Invitrogen) FITC-conjugated antibodies, and samples were injected into the flow cytometer device. Flow cytometry was performed using a Novocyte D3005 (Agilent) standard configuration. The data were analysed with flow cytometry software (version 1.5.0, Agilent).

#### 2.3.1 *In vitro* Uptake Analysis of hSF-MSC-Exos

To characterize functionally isolated exosomes, *in vitro* uptake analysis was performed. Briefly, exosomes labelled with 1µM of PKH26, a lipophilic red fluorescent dye, by using the PKH26 Red Fluorescent Cell Linker Kit for the general cell membrane labelling kit (Sigma-Aldrich) to observe their uptake by MSCs according to the manufacturer’s protocols. 100 µl of diluent C was added to the exosomes in PBS (Gibco) containing 1µl of PKH26 dye. Exosome and dye samples were prepared and mixed gently for 4 minutes; excess dye was removed using PBS.

PKH26 labeled exosomes were co-cultured with hSF-MSCs to observe their uptake by MSCs. Cells were observed and photographed before and after 3 hours of co-culture using a fluorescent microscope (Axio Observer D1, Carl Zeiss, Oberkochen, Germany).

### 2.4 Chondrogenic Differentiation from Fetal Human Chondroblasts (hFCs) to Mature Human Chondrocytes (hFCs)

Human fetal chondroblast cell line (hFC) were chondrogenically differentiated to obtain control group human mature chondrocytes (hMCs). hFC line was purchased from Sigma Aldrich. Cells were thawed and cultured in a complete culture medium, DMEM (Gibco), containing 10% FBS (Gibco) and 100 IU/ml penicillin (Gibco), 100 µg/ml streptomycin (Gibco). To obtain hMCs, cells were cultured in a chondrogenic differentiation medium consists of high glucose DMEM (Gibco) supplemented with 10 ng/ml TGF-β1 (Sigma Aldrich) 50 µg/ml ascorbate-2-phosphate (Wako Chemicals), 0.1 µM dexamethasone (Sigma Aldrich), 100 µg/ml sodium pyruvate (Sigma Aldrich), 40 µg/ml proline (Merck), 50 mg/ml ITS premix (BD Biosciences), and 0.2% primocin (Invivogen) for two weeks.

### 2.5 Quantitative Gene Expression Analysis by RT-qPCR

In the first step the expression levels of hsa-miR-155-5p, some TGF-β signaling pathway genes, ACAN, and Coll IA2 in hSF-MSCs compared to them in the exosomes. In the second step, the expression levels of the same miR and genes in chondroblasts differentiated from hSF-MSCs were compared with those of mature human chondrocytes. The relative gene expression levels of the ACAN, Coll IA2, TGF-1, SMAD2, SMAD3, and SMAD4 genes involved in the TGF-β signaling pathway were examined using RT-qPCR (QIAGEN Rotor-GeneQ, Hilden, Germany). Probe primers for each target gene are mentioned in Table **2**. Additionally, the relative expression of the microRNA, hsa-miR-155-5p, is needed to validate its role in the chondrogenesis process. For the production of cDNA, previously collected mRNA samples from experiment groups were used. The A.B.T.^TM^ cDNA synthesis kit (dT20) (Atlas Biotechnology, Ankara, Turkey) methodology was used when creating cDNA from mRNA samples. The cDNA synthesis for miRNA samples was applied according to the same protocol as the A.B.T.™ cDNA synthesis kit with the use of suitable primers for hsa-miR-155-5p and U6 small nuclear RNA. (Hsa-miR-155-5p: GAAAGAAGGCGAGGAGCAGAT CGAGGAAGAAGACGGAAGAATGTGCGTCTCGCCTTCTTTCACCCCTAT), and (U6: CGCTTCACGA ATTTGCGTGTCAT). The generated cDNA samples from each group were used for the RT-qPCR reaction once the cDNA synthesis reaction was completed. This reaction was carried out using RealTime AMPLIFYME SYBRTM Green No-ROX Mix (Blirt, Poland, AM01). The manufacturer's procedure was followed during the protocol. Both the housekeeping genes GAPDH and U6 were employed as control genes in our RT-qPCR experiment.

Using Microsoft Excel, the relative expression or fold change of each gene and miRNA in hSF-MSCs, hSF-MSC-Exo, hSF-MSCs that have undergone chondrogenically differentiated (hSF-MSC-chondro), and hMCs was represented separately.

### 2.6 Statistical Analysis

All the graphs, calculations, and statistical analyses were performed using GraphPad Prism software version 9.2 using one simple t test and multiple t tests. p values were considered statistically significant if the p value was less than or equal to 0.05 (*p≤0.05, **p≤0.01, ***p≤0.001, ****p≤0.000). Each experiment was repeated at least three times.

## 3 RESULTS

Isolated hSF-MSCs were propagated and cultured until they reached the third passage, and then they were taken for stem cell characterization analyses and experiments. During the initial days of incubation, the cultured cells showed sparse adherence to the culture flasks and displayed a spindle-shaped morphology resembling fibroblasts. Subsequently, after about three days, the cells started to proliferate and gradually formed small colonies (Fig. **1A**). Seven days after their first passage, the cultured cells attained monolayer confluence in the primary culture. In the subsequent passages, the majority of the cells displayed a flattened or fibroblast-like morphology (Fig. **1B**). In immunophenotypic characterization results supported by flow cytometry results (Fig. **1C**), hSF-MSCs were found positive for CD105 (99,13%), CD90 (100,00%), CD73 (100,00%), CD44 (100,00%), MHC I (99.65%), CD29 (99,98%), CD45 (0,00%) (Fig. **2A**), MHC II (0.03%) (Fig. **2B**), FIBRONECTIN (Fig. **2C**), C-FOS (Fig. **2D**), NESTIN (Fig. **2E**), α-SMA (Fig. **2F**), ACTIN and negative for CD69 (5,41%), CD34 (0,11%) and CD25 (0,03%) markers.

**Fig. (3):**
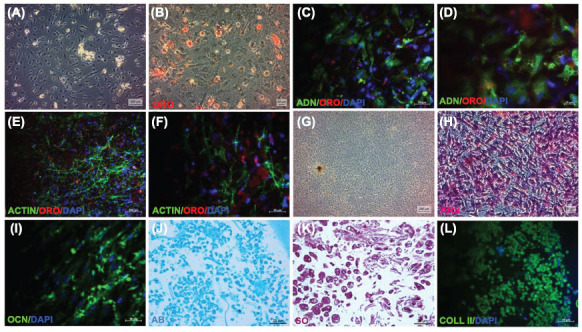
Photomicrographs of hSF-MSCs after differentiation induction. (**A**): The phase-contrast microscope appearance of hSF-MSCs differentiated into an adipogenic lineage after 15 days. Histo- and/or immunohistochemical techniques that detected adipo-specific markers were applied to cells grown in adipogenic differentiation medium to assess the adipogenic differentiation of hSF-MSCs. B and F: Lipid droplets were positive stained with ORO (**B**, **E** and **F**). Cytoskeleton was positive with ACTIN (**E** and **F**), and adipogenic differentiated cells were positive with ADN (**C** and **D**). G: After osteogenic differentiation for 28 days, clustered spiculinous calcium deposits were characterized by nodular aggregates at phase contrast microscope. (**H** and **I**): Mineral nodules were stained positively with ARS and OCN respectively. **J-L**: After four weeks of the chondrogenic differentiation cells were positive stained with AB (**J**) and SO (**K**) for histochemical and positive labelled with COLL II (**L**) for immunohistochemical analysis. Scale bars: (**G**) 200 μm; (**A** and **E**) 100 μm; (**B**, **C**, **F**, **H**, **I** and **L**) 50 μm; (**D**, **J**, and **K**) 20 μm.

The morphologic, histologic (Fig. **1A** and **B**), and immunohistochemical characteristics were analysed in the differentiation characterization. At the end of the third week of the adipogenic differentiation, lipid droplets were observed in the differentiated cells at phase contrast microscope (Fig. **3A**). Lipid droplets in adipogenic differentiated cells positively stained with Oil Red O dye (Fig. **3B**-**F**). These cells were positive for ADN (Fig. **3C** and **D**) and Table **1**. ORO was double-labelled with cytoplasmic ACTIN to show the positive lipid droplets intra or extracellular more representatively (Fig. **3E** and **F**). After osteogenic differentiation cultures for 28 days, clustered spiculinous calcium deposits characterized by nodular aggregates under a phase contrast microscope. The morphology changed from fibroblast-like to multilayer and spherical form (Fig. **3G**). The nodular aggregates in osteogenically differentiated cells histochemically stained with Alizarin Red S confirmed osteogenic differentiation (Fig. **3H**). The cells also immuno-cytochemically labelled positive for OCN (Fig. **3I** and Table **1**).

**Fig. (4):**
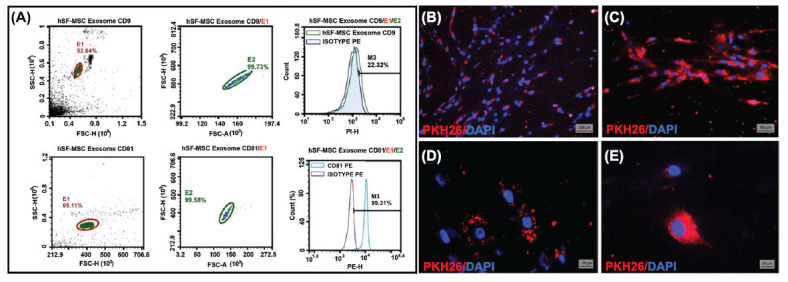
Phenotypic (**A**) and functional (**B**-**E**) characterization of exosomes isolated from hSF-MSC by flow cytometry and cell uptake analyses. (**A**): Isolated exosomes were found to be positive for exosome-specific CD9, and CD81 markers. (**B**): Extracellular exosomes observed outside the cells at the beginning of the co-culture of exosomes with hSF-MSCs. (**C**-**E**): Exosomes uptake by hSF-MSCs were shown with PKH26 (red) after 3 hours of co-culture. Cell nuclei were labelled with DAPI dye (blue). Scale bars: (**B**) 100 µm; (**C**) 50 µm; (**D**) and (**E**) 20 µm.

After four weeks of chondrogenic differentiation, cells positively stained with AB (Fig. **3J**) and SO (Fig. **3K**) dyes for histochemical analysis and positively labelled with COLL II (Fig. **3L**) and Table **1** for immuno histochemical analysis. Isolated exosomes from hSF-MSC were found to be positive for exosome-specific CD9 (22.32%), and CD81 (99.31%) surface markers in flow cytometry analysis (Fig. **4A**). hSF-MSC-Exos labelled with PKH26 were taken up by hSF-MSCs, after they co-cultured with hSF-MSCs. The exosome uptake time was 3 hours. Uptaken exosomes localized in the cytoplasmic regions surrounding the nucleus (Fig. **4B**-**E**).

**Fig. (5):**
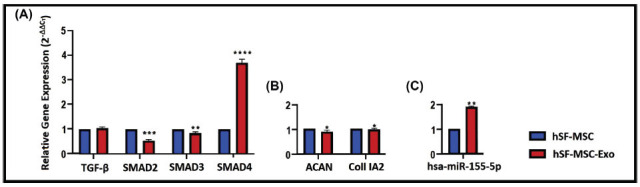
(**A**): Expression profile of TGF-β signalling pathway genes in hSF-MSC and hSF-MSC-Exo groups. TGF-β gene expression level were not changed in hSF-MSC-Exo compared to hSF-MSC group. In hSF-MSC-Exo group both Smad2 and Smad3 expression levels were decreased compared to the hSF-MSC group while Smad4 was increased. (**B**): ACAN and Coll IA2 gene expression profiles in same groups. In hSF-MSC-Exo group, ACAN and Coll IA2 gene expression levels was decreased compared to the hSF-MSCs group (**C**): Micro RNA hsa-miR-155-5p expression levels in same groups. In hSF-MSC-Exo group hsa-miR-155-5p expression was increased compared to the hSF-MSC group (n=3, *p≤0.05, **p≤0.01, ***p≤0.001, ****p≤0.000).

The TGF-β gene expression level in the exo group isolated from hSF-MSCs was nearly identical to that of their parents, the hSF-MSC group. However, in the hSF-MSC-Exo group, both Smad2 (***p≤0.001) and SMAD3 (**p≤0.01) expression levels decreased compared with the hSF-MSC group, while Smad4 significantly increased (****p≤0.000) (Fig. **5A**). In the hSF-MSC-Exo group, ACAN and Coll IA2 gene expression levels decreased compared to the hSF-MSCs group (*p≤0.05) (Fig. **5B**). hsa-miR-155-5p expression level increased in the hSF-MSC-Exo group compared with the hSF-MSC (**p≤0.01) (Fig. **5C**).

**Fig. (6):**
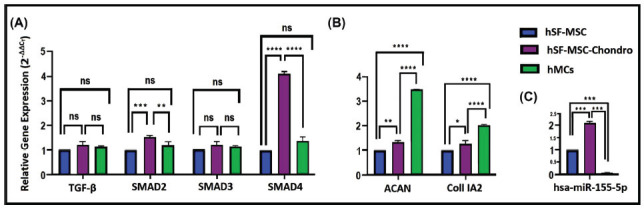
Expression profile of TGF-β signalling pathway genes in hSF-MSC-chondro and hMCs groups. (**A**): Smad2 and Smad4 gene expression levels increased in hSF-MSC-chondro compared with the hMCs. (**B**): ACAN and Coll IA2 gene expression profiles in hSF-MSC-chondro and hMCs groups. ACAN and Coll IA2 gene expression levels were decreased in hSF-MSC-chondro compared with the hMCs groups. In addition to same gene expression levels were increased in differentiated hSF-MSC-chondro compared with hSF-MSCs. (**C**): Micro RNA hsa-miR-155-5p expression levels in hSF-MSC-chondro and hMCs groups. hsa-miR-155-5p expression level was increased in the hSF-MSC-chondro compared with hMCs group. Similarly, hsa-miR-155-5p expression levels were increased in differentiated hSF-MSC-chondro compared with hSF-MSCs (n=3, *p≤0.05, **p≤0.01, ***p≤0.001).

SMAD2 and SMAD4 gene expression levels increased in hSF-MSC-chondro compared with the hMCs (**p≤0.01). TGF-β and SMAD3 gene expression levels also increased but non-significantly. Additionally, SMAD2 and SMAD4 gene expression levels increased in differentiated hSF-MSC-chondro compared with the hSF-MSC group (Fig. **6A**). ACAN and Coll IA2 gene expression levels decreased in hSF-MSC-chondro compared with the hMCs (*p≤0.05). In addition, the same gene expression levels increased in differentiated hSF-MSC-chondro compared with hSF-MSCs (Fig. **6B**). The hsa-miR-155-5p expression level increased in the hSF-MSC-chondro group (*p<0.05), while it decreased in the hMCs group. Similarly, the hsa-miR-155-5p expression levels increased in differentiated hSF-MSC-chondro compared with hSF-MSCs (Fig. **6C**). The experiments showed a correlation between SMAD2 and SMAD4 gene expression levels and miR-155-5p in differentiated hSF-MSC-chondro compared with the hSF-MSC group. SMAD2, SMAD4 and hsa-miR-155-5p expression level increased in the hSF-MSC-chondro group compared with the undifferentiated stem cell control group (Fig. **6A** and **6C**). About the results of the hMCs group; SMAD2, SMAD3 and SMAD4 gene expression showed a non-significant change in the hMC group compared to the hSF-MSC group, while the hsa-miR-155-5p expression level decreased (Fig. **6A** and **6C**).

## 4 DISCUSSION

This study designed for the purpose of contributing to cartilage tissue regeneration research to develop a safe cell-free clinical approach and as their source synovial fluid for treating and repairing damaged cartilage tissue. Accordingly, the expression levels of hsa-miR-155-5p and its target genes of TGF-β signaling pathway SMAD2, SMAD3, and SMAD4 were investigated in hSF-MSCs and in hSF-MSCs-exo’s. RT-qPCR was used to examine the expression levels of the TGF-β signaling pathway and hsa-miR-155-5p, and the results were compared with those from experimental groups. Exosomes are a great candidate to be employed as drug delivery vehicles because of their function in controlling intercellular communication, which enables them to be loaded with therapeutic compounds [12, 13, 25, 26]. Investigating micro RNAs (miRNAs), which may target both the chondrogenic differentiation process and the pathogenesis of cartilage injury, is another crucial aspect of treating cartilage damage. miRNAs are a class of non-coding RNAs that play important roles in RNA silencing as well as in post-transcriptional regulation of gene expression. It is anticipated that miRNAs play a pivotal role in the development of many diseases. In a study on the effect of miRNA on the chondrogenesis process, it was determined that the dicer endonuclease enzyme activity has a prominent role in the chondrogenesis process by participating in its biogenesis [27]. Tao *et al*.'s study [28], which transfected miR-140 into exosomes isolated from MSCs produced from synovial fluid and examined chondrocyte cell proliferation and migration, indicated the function of miR-140 in preventing osteoarthritis. In researches, bone marrow-derived MSCs are the most often employed form of MSCs. However, bone marrow harvesting is an invasive and painful procedure that can cause morbidity. In addition, MSCs isolated from bone marrow constitute only 0.01% of the bone marrow cells [29]. It has therefore become important to obtain a better and less invasive source of MSC. For example, isolating MSCs from synovial fluid is a less invasive alternative and the isolated cells are multipotent.

In this study, we isolated MSCs from hSF-MSC. Following the propagation of isolated cells under standard cell culture conditions, characterization tests were carried out. In order to identify particular stem cell surface markers in isolated hSF-MSCs, immunophenotypic analyses were carried out using flow cytometric analysis. According to our findings, hSF-MSCs were positive for CD105, CD90, CD73, CD44, MHC I, CD29, FIBRONECTIN, C-FOS, NESTIN, α-SMA, ACTIN and negative for CD69, CD45, CD34, CD25 and MHC II markers. We were able to successfully differentiate the isolated hSF-MSCs from our investigation into the adipogenic, osteogenic, and chondrogenic cell lineages. It was observed that oil droplets were stained with ORO dye in adipogenic differentiated cells and ADN was positive. Nevertheless, calcium nodules were stained with ARS dye and OCN marker was found to be positive in osteogenic differentiated cells. The extracellular matrix containing glycosaminoglycans was positively stained purple by SO, indicating that the observed morphology of chondrogenic differentiated cells was similar to that of chondroblasts where lacunae were observed. This indicates that cells have positively differentiated into the chondrogenic lineage. Our observed results suggest that isolated hSF-MSCs have demonstrated the minimal features to be identified as MSCs, in accordance with the International Society for Cellular Therapy (ISCT) standard statement for identifying MSCs. In our investigation, we isolated exosomes from hSF-MSCs and characterized them using an immunophenotypic examination of exosome-specific surface markers. They were shown that the expression of CD9 and CD81 exosome-specific surface markers indicated exosomes [30-32].

The goal of the current investigation was to determine whether using hSF-MSC and their exosomes as a safer therapeutic option for cartilage damage could be accomplished by analyzing the expression levels of hsa-miR-155-5p and TGF-β signaling pathway genes. The miRTarBase database (https://mirtarbase.cuhk.edu.cn/~miRTarBase/miRTarBase_2022/php/search.php) reveals that the genes SMAD2, SMAD3, and SMAD4 are hsa-miR-155-5p target genes. Additionally, they support the initial stages of chondrogenic differentiation through their participation in the TGF-β signaling pathway [15, 18, 19]. Therefore, in addition to examining the expression of hsa-miR-155-5p, we also examined the expression of the TGF-β signaling pathway genes SMAD2, SMAD3, and SMAD4 as well as the expression of the chondrogenic differentiation process markers Coll IA2 and ACAN in order to advance research on cartilage tissue regeneration and create a secure cell-free clinical strategy to treat and restore damaged cartilage tissue.

In the present study, hsa-miR-155-5p expression level in hSF-MSC-Exo was increased compared to the parent’s group (hSF-MSC), and a similar expression level was observed between hSF-MSC and their exosomes in the expression level of TGF-β, which has a main role in chondrogenic differentiation. In hSF-MSC-Exo group, ACAN and Coll IA2 gene expressions were decreased compared to the hSF-MSCs group. The expression of SMAD proteins was also significantly different between hSF-MSCs and their exosomes. This confirms that we could use the exosome secreted by the cell instead of the cell itself in cell-based therapies, which is more advantageous. Moreover, Coll IA2, and ACAN gene expression levels by RT-qPCR and compared to control group hMCs differentiated from commercially purchased hFCs. In hSF-MSC-chondro, Coll IA2 level was decreased according to control group hMCs. At both the early and advance stages of the chondrogenic differentiation process, chondrocyte cells are known to produce the Coll IA2 gene. This was supported by the results from both our study's hMCs, which are at an advanced state of chondrogenic differentiation, and hSF-MSC-chondro, which represents early differentiated cells. Proteoglycan, a crucial element of the extracellular matrix of cartilage tissues, is synthesized by the ACAN gene. In our investigation, it was discovered that hSF-MSC-chondro had lower levels of ACAN gene expression than hMCs, which is consistent with the notion that cartilage tissue and chondrogenic differentiated cells primarily express the ACAN gene.

Kolhe *et al*.'s study [14] is one of the studies that investigated miRNAs and exosomes obtained from synovial fluid to show whether they play a role in the pathogenesis of osteoarthritis in both sexes or not. In this study, sex-specific exosomal miRNAs and their target signaling pathways were investigated, and it was discovered that miRNAs were implicated in osteoarthritis. miR-155, which was estrogen sensitive and targeted TLR signaling pathways in females, was revealed to be involved in osteoarthritis [14]. One of the miRNA subgroups known to modulate innate immune response *via* controlling inflammation is miR-155, which is stimulated by pro-inflammatory molecules like TNF-α and IL-1β [33]. Another study examining the impact of miRNAs in MSCs and osteoarthritis found that upregulating miR-155 expression decreased MSCs' the immunosuppressive capacity. Additionally, they claimed that utilizing MSCs obtained from adipose tissue improved the process of chondrogenesis by lowering macrophage activation in the treatment of osteoarthritic cells [34]. miR-155 is a multifunctional miRNA that has significant roles in the innate and adaptive immune response in arthritis and may be a possible biomarker for rheumatoid arthritis disease, according to research by Alivernini *et al*. [35]. We found that hsa-miR-155-5p expression in the hSF-MSC-Exos was increased compared to hSF-MSCs. While it was up-regulated in the hSF-MSC-chondro group, we deduced that this group of cells had not completed their chondrogenic differentiation; therefore, their expression was continuing. Our results showed that isolated exosomes from hSF-MSCs were found to be positive for exosome-specific surface markers in flow cytometry analysis. hSF-MSCs exosomes labelled with PKH26 were uptaken as the result of co-culture with hSF-MSCs. Exosome uptake time was 3 hours. Uptake exosomes were localized in the cytoplasmic areas around the nucleus.

A few studies have been undertaken to demonstrate the function of the TGF-β signaling pathway, particularly in the chondrogenic differentiation of bone marrow-derived MSCs, in the chondrogenesis process during embryonic development. De Kroon *et al*. conducted one of these investigations, demonstrating the function of the SMAD2, SMAD3, and SMAD4 genes in TGF-β-induced chondrogenesis in bone marrow-derived MSCs [36]. Researchers found that TGF-β-induced chondrogenesis in bone marrow-derived MSCs is mediated by SMAD3 and SMAD4 genes. They found that overexpressing or knocking down the SMAD3 gene fully inhibited TGF-β-induced chondrogenesis, whereas modifying the expression of the SMAD2 gene had little impact on chondrogenesis [36, 37]. Upon completion of chondrogenesis, MSC-derived chondrocytes may display signs of hypertrophic differentiation, which is not ideal for the formation of articular cartilage as hypertrophic chondrocytes produce cartilage that will mineralize and ossify when implanted *in vivo*. In a study by de Kroon *et al*., it was found that modulating SMAD2 expression did not have an effect on the expression of hypertrophic differentiation markers, nor did SMAD4 overexpression. Our findings indicated that an increase in TGF-β gene expression, which plays a major role in chondrogenic differentiation, in the hSF-MSC-chondro group compared to hMCs. The expression level of SMAD proteins involved in the TGF-β signaling pathway also increased. The increase in TGF-β expression in the hSF-MSC-chondro group can be attributed to the fact that the chondrogenic differentiation process of these cells is still ongoing and they are in the stage between condensation and full differentiation/maturation. This finding is supported by literature and our finding demonstrated that expression levels of ACAN and COL1A2 maintained in hSF-MSC-chondro, more than in hSF-MSC but less than in hMCs [38]. In this study, significantly increase in expression levels of SMAD2 and SMAD4 supported that hSF-MSC-chondro was at maturation stage not at hypertrophy stage. The lower expression levels of TGF-β and SMAD 2/3/4 genes compared with hSF-MSC-chondro prove that hMCs are fully differentiated cells. In mammary epithelial cells of mice, TGF-β is known to stimulate the promoter activity of hsa-miR-155-5p expression *via* SMAD4 [39]. In this study, hsa-miR-155-5p expression was increased in the group of hSF-MSC-chondro compared to hMC group which is related to the increased expression levels of TGF-β, SMAD 2/3/4 signalling pathway genes and decreased expression levels of ACAN and COL1A2 during chondrogenic differentiation from MSCs. We hypothesize that it is because the chondrogenic differentiation process has not been fully finished and because the expression of hsa-miR-155-5p is linked to EMT, cell proliferation, and migration. Runx2, which is also implicated in bone differentiation, is a target of hsa-miR-155-5p, which is known to promote ECM secretion [40]. In this study, induced hsa-miR-155-5p expression has been shown to maintain cell proliferation and secretion of ECM indicated with increased mature chondrocyte phenotype compared stem cells while reduced immature chondrocyte phenotype (ACAN and COL1A2) in hSF-MSC-chondro group compared to hMCs group. Our findings support hsa-miR-155-5p's role in chondrogenesis by showing that it can boost Runx2-mediated ECM production, which occurs during the complete differentiation/maturation stage of chondrogenic differentiation.

This study presents the results of a pivotal preliminary investigation into stem cell-based or cell-free gene therapy. In order for this treatment to be applied in cases of cartilage damage, the target gene or miRNA must first be determined. This study was conducted to ascertain the target genotype before applying gene therapy for cartilage damage. Furthermore, it explores the potential of exosomes as natural organic carriers capable of delivering the targeted genotype for genotypic treatment of chondrocyte damage. Moreover, it suggests that obtaining exosomes from MSCs isolated from joint synovial fluid, which is closely related to cartilage tissue, may enhance targeting. While our study provides initial evidence that miR-155-5p can be used as a target miRNA and exosomes as carriers in a novel gene therapy approach, there are still missing points. To address these gaps, future research should involve transferring miR-155-5p to SF-MSCs, isolating exosomes from these cells, and investigating the effects of these exosomes *in vitro* using a chondrocyte injury model and *in vivo* through an animal model with cartilage damage. This comprehensive approach will help elucidate the potential of miR-155-5p and exosomes in gene therapy for cartilage damage, moving us closer to the development of an effective and promising treatment strategy.

## CONCLUSION

In the hSF-MSC-chondro group, it has been discovered through this work that the enhanced expression of hsa-miR-155-5p is connected to the increased expression of SMAD 2 and 4 genes. Furthermore, we observed that hsa-miR-155-5p and SMAD 4 expression level was significantly higher in hSF-MSC-Exo compared to its expression in hSF-MSCs. It was concluded that human synovial fluid derived MSCs and their exosomes can be used in the treatment of cartilage damage. Moreover, this study indicated the role of hsa-miR-155-5p in chondrogenesis that could be used in a gene therapy combined with hSF-MSCs along with their exosomes as a cell-based or -free gene therapy option for cartilage damage. Future research on the variation in hsa-miR-155-5p expression levels in different stages of the chondrogenic differentiation process will undoubtedly be supported by these results. As a result, in order to comprehend and better explain the process of chondrogenesis, we also recommend elucidating other related signaling pathways and their receptors.

## Figures and Tables

**Table 1:** Immunophenotypic characteristics and differentiation characterization of hSF-MSCs.

**Marker**	**Antibody Dilution**	**Marker Expression**
CD105	1:100	99,13%
CD90	1:100	100,00%
CD73	1:100	100,00%
CD44	1:100	100,00%
MHC CLASS I	1:100	99.65%
CD29	1:100	99,98%
CD69	1:100	5,41%
CD45	1:100	0,00%
CD34	1:100	0,11%
CD25	1:100	0,03%
MHC CLASS II	1:200	0.03%
FIBRONECTIN	1:100	+
NESTIN	1:100	+
C-FOS	1:50	+
α-SMA	1:100	+
ADIPONECTIN	1:200	+
OSTEOCALCIN	1:50	+
COLLAGEN II	1:50	+

**Table 2:** Target gene primers.

**Gene**	**Sequences**		**Size (bp)**	**Tm (°C)**
GAPDH	F	TCGGAGTCAACGGATTTGGT	20	59.32
	R	TTCCCGTTCTCAGCCTTGAC	20	59.97
COL1A2	F	CTGGTCTCGGTGGGAACTTT	20	59.60
	R	ACCCTGTGGTCCAACAACTC	20	59.82
TGFβ	F	TACCTGAACCCGTGTTGCTC	20	59.97
	R	CCGGTAGTGAACCCGTTGAT	20	59.75
SMAD2	F	TGTTTTCAGTTCCGCCTCCA	20	59.82
	R	AGCCTCTTGTATCGAACCTGC	21	60.13
SMAD3	F	GTGCTGGGGTTAGGTCACTG	20	60.32
	R	CTGTGGGAATGTCGCATCCT	20	60.11
SMAD4	F	TAACGGAGCGGTTTGGGTG	19	60.30
	R	TTCAGGATAACCTGGGCTCG	20	59.17
ACAN	F	CAAACGCAGACTACAGAAGCG	21	59.87
	R	TGATTTGGAGGGGTGAGTGG	20	59.30
U6	F	GCTTCGGCAGCACATATACTAAAAT	25	54.40
	R	CGCTTCACGAATTTGCGTGTCAT	23	55.27

**F:** Forward; **R:** Reverse.

## Data Availability

The data used to support the findings of this study are available from the corresponding author upon request. In addition, since this study is derived from a master's thesis, all kinds of resources can be accessed online from from the following link. file:///C:/Users/user/Downloads/689089.pdf.

## LIST OF ABBREVIATIONS

AB: Alcian Blue
ACAN: Aggrecan
ADN: Adıponectin
ARS: Alizarin Red S
COL1A2: Collagen Alpha-2(I) Chain
DAPI: Diamidino-2-phenylindole
DMEM: Dulbecco's Modified Eagle's Medium
EDTA: Ethylenediamine Tetraacetic Acid
FBS: Fetal Bovine Serum
FITC: Fluorescein İsothiocyanate
hFC: Chondroblast Cell Line
hMCs: Human Mature Chondrocytes
hSF-MSC: Human Synovial Fluid-derived Mesenchymal Stem Cell
IBMX: Isobutylmethylxanthine
IF: Immunofluorescence Labelling
OCN: Osteocalcin
ORO: Oil Red O
RT-qPCR: Reverse Transcription Polymerase Chain Reaction
SC: Stem Cell
SO: Safranin O
TGF: Transforming Growth Factor

## References

[1] Zhao W., Wang T., Luo Q. (2016). Cartilage degeneration and excessive subchondral bone formation in spontaneous osteoarthritis involves altered TGF-β signaling.. J. Orthop. Res..

[2] Hosseini S., Taghiyar L., Safari F., Baghaban Eslaminejad M. (2018). Regenerative medicine applications of mesenchymal stem cells.. Adv. Exp. Med. Biol..

[3] Loo S., Wong N. (2021). Advantages and challenges of stem cell therapy for osteoarthritis (Review).. Biomed. Rep..

[4] Chang C., Yan J., Yao Z., Zhang C., Li X., Mao H.Q. (2021). Effects of mesenchymal stem cell‐derived paracrine signals and their delivery strategies.. Adv. Healthc. Mater..

[5] Mirotsou M., Jayawardena T.M., Schmeckpeper J., Gnecchi M., Dzau V.J. (2011). Paracrine mechanisms of stem cell reparative and regenerative actions in the heart.. J. Mol. Cell. Cardiol..

[6] Wei W., Ao Q., Wang X. (2021). Mesenchymal stem cell–derived exosomes: A promising biological tool in nanomedicine.. Front. Pharmacol..

[7] Ferguson S.W., Nguyen J. (2016). Exosomes as therapeutics: The implications of molecular composition and exosomal heterogeneity.. J. Control. Release.

[8] Nikfarjam S., Rezaie J., Zolbanin N.M., Jafari R. (2020). Mesenchymal stem cell derived-exosomes: A modern approach in translational medicine.. J. Transl. Med..

[9] Lai R.C., Yeo R.W.Y., Tan K.H., Lim S.K. (2013). Exosomes for drug delivery-a novel application for the mesenchymal stem cell.. Biotechnol. Adv..

[10] Shang X., Fang Y., Xin W., You H. (2022). The application of extracellular vesicles mediated mirnas in osteoarthritis: Current knowledge and perspective.. J. Inflamm. Res..

[11] Baglio S.R., Pegtel D.M., Baldini N. (2012). Mesenchymal stem cell secreted vesicles provide novel opportunities in (stem) cell-free therapy.. Front. Physiol..

[12] Biancone L., Bruno S., Deregibus M.C., Tetta C., Camussi G. (2012). Therapeutic potential of mesenchymal stem cell-derived microvesicles.. Nephrol. Dial. Transplant..

[13] Zacharias E., Milton G., Momen-Heravi F., Hu J., Zhang X., Wu Y. (2013). Therapeutic uses of exosomes.. J. Circ. Biomark..

[14] Kolhe R., Hunter M., Liu S. (2017). Gender-specific differential expression of exosomal miRNA in synovial fluid of patients with osteoarthritis.. Sci. Rep..

[15] Iaquinta M.R., Lanzillotti C., Mazziotta C. (2021). The role of microRNAs in the osteogenic and chondrogenic differentiation of mesenchymal stem cells and bone pathologies.. Theranostics.

[16] Xu C., Ren G., Cao G. (2013). miR-155 regulates immune modulatory properties of mesenchymal stem cells by targeting TAK1-binding protein 2.. J. Biol. Chem..

[17] Pers Y.M., Bony C., Duroux-Richard I. (2021). miR-155 contributes to the immunoregulatory function of human mesenchymal stem cells.. Front. Immunol..

[18] Wang W., Rigueur D., Lyons K.M. (2014). TGFβ signaling in cartilage development and maintenance.. Birth Defects Res. C Embryo Today.

[19] van der Kraan P.M., Blaney Davidson E.N., Blom A., van den Berg W.B. (2009). TGF-beta signaling in chondrocyte terminal differentiation and osteoarthritis.. Osteoarthritis Cartilage.

[20] Sariboyaci A., Uysal O., Gunes S. (2021). Differentiation of human bone marrow-derived mesenchymal stem cells into functional pancreatic beta cells.. Med. Sci..

[21] Duman B.O., Sariboyaci A.E., Karaoz E. (2022). Bio-engineering of 3-D cell sheets for diabetic rats: Interaction between mesenchymal stem cells and beta cells in functional islet regeneration system.. Tissue Cell.

[22] Demircan P.C., Sariboyaci A.E., Unal Z.S., Gacar G., Subasi C., Karaoz E. (2011). Immunoregulatory effects of human dental pulp-derived stem cells on T cells: Comparison of transwell co-culture and mixed lymphocyte reaction systems.. Cytotherapy.

[23] Karaöz E., Doğan B.N., Aksoy A. (2010). Isolation and *in vitro* characterisation of dental pulp stem cells from natal teeth.. Histochem. Cell Biol..

[24] Sariboyaci A., Demircan P., Gacar G., Unal Z., Erman G., Karaoz E. (2014). Immunomodulatory properties of pancreatic islet-derived stem cells co-cultured with T cells: Does it contribute to the pathogenesis of type 1 diabetes?. Exp. Clin. Endocrinol. Diabetes.

[25] Zhou Y., Xu H., Xu W. (2013). Exosomes released by human umbilical cord mesenchymal stem cells protect against cisplatin-induced renal oxidative stress and apoptosis *in vivo* and *in vitro*.. Stem Cell Res. Ther..

[26] Zhang R., Ma J., Han J., Zhang W., Ma J. (2019). Mesenchymal stem cell related therapies for cartilage lesions and osteoarthritis.. Am. J. Transl. Res..

[27] Pan Y., Balazs L., Tigyi G., Yue J. (2011). Conditional deletion of Dicer in vascular smooth muscle cells leads to the developmental delay and embryonic mortality.. Biochem. Biophys. Res. Commun..

[28] Tao S.C., Yuan T., Zhang Y.L., Yin W.J., Guo S.C., Zhang C.Q. (2017). Exosomes derived from miR-140-5p-overexpressing human synovial mesenchymal stem cells enhance cartilage tissue regeneration and prevent osteoarthritis of the knee in a rat model.. Theranostics.

[29] Mao G., Zhang Z., Hu S. (2018). Exosomes derived from miR-92a-3p-overexpressing human mesenchymal stem cells enhance chondrogenesis and suppress cartilage degradation *via* targeting WNT5A.. Stem Cell Res. Ther..

[30] Melo S.A., Luecke L.B., Kahlert C. (2015). Glypican-1 identifies cancer exosomes and detects early pancreatic cancer.. Nature.

[31] Xu R., Simpson R.J., Greening D.W. (2017). A protocol for isolation and proteomic characterization of distinct extracellular vesicle subtypes by sequential centrifugal ultrafiltration Exosomes and Microvesicles..

[32] Chen J., Chen J., Cheng Y. (2020). Mesenchymal stem cell-derived exosomes protect beta cells against hypoxia-induced apoptosis *via* miR-21 by alleviating ER stress and inhibiting p38 MAPK phosphorylation.. Stem Cell Res. Ther..

[33] Swingler T.E., Wheeler G., Carmont V. (2012). The expression and function of microRNAs in chondrogenesis and osteoarthritis.. Arthritis Rheum..

[34] Pers Y.M., Ruiz M., Noël D., Jorgensen C. (2015). Mesenchymal stem cells for the management of inflammation in osteoarthritis: State of the art and perspectives.. Osteoarthritis Cartilage.

[35] Alivernini S., Gremese E., McSharry C. (2018). MicroRNA-155—at the critical interface of innate and adaptive immunity in arthritis.. Front. Immunol..

[36] de Kroon L.M.G., Narcisi R., van den Akker G.G.H. (2017). SMAD3 and SMAD4 have a more dominant role than SMAD2 in TGFβ-induced chondrogenic differentiation of bone marrow-derived mesenchymal stem cells.. Sci. Rep..

[37] Green J.D., Tollemar V., Dougherty M. (2015). Multifaceted signaling regulators of chondrogenesis: Implications in cartilage regeneration and tissue engineering.. Genes Dis..

[38] Guidotti S., Minguzzi M., Platano D. (2017). Glycogen synthase kinase-3β inhibition links mitochondrial dysfunction, extracellular matrix remodelling and terminal differentiation in chondrocytes.. Sci. Rep..

[39] Kong W., Yang H., He L. (2008). MicroRNA-155 is regulated by the transforming growth factor β/Smad pathway and contributes to epithelial cell plasticity by targeting RhoA.. Mol. Cell. Biol..

[40] Wang Z., Yan K., Ge G. (2021). Exosomes derived from miR-155-5p–overexpressing synovial mesenchymal stem cells prevent osteoarthritis *via* enhancing proliferation and migration, attenuating apoptosis, and modulating extracellular matrix secretion in chondrocytes.. Cell Biol. Toxicol..

